# Consolidative treatment after salvage chemotherapy improves prognosis in patients with relapsed extranodal natural killer/T-cell lymphoma

**DOI:** 10.1038/srep23996

**Published:** 2016-04-04

**Authors:** Man Nie, Xi-wen Bi, Wen-wen Zhang, Peng Sun, Yi Xia, Pan-pan Liu, Hui-qiang Huang, Wen-qi Jiang, Zhi-ming Li

**Affiliations:** 1 Department of Medical Oncology, Sun Yat-sen University Cancer Center, Guangzhou, 510060, P. R. China; 2 Department of Radiation Oncology, Sun Yat-sen University Cancer Center, Guangzhou, 510060, P. R. China; 3 State Key Laboratory of Oncology in South China, Collaborative Innovation Center for Cancer Medicine, Guangzhou, 510060, P. R. China

## Abstract

The optimal treatment strategy for relapsed natural killer/T-cell lymphoma (NKTCL) remains largely unknown. We retrospectively reviewed the treatment modalities and prognosis of 56 relapsed NKTCL patients. Chemotherapy was the initial salvage treatment, followed by radiotherapy (RT) or autologous hematopoietic stem cell transplantation (AHSCT) as consolidative therapy, depending on the status of remission and the pattern of relapse. For patients with locoregional relapse alone, consolidative RT after salvage chemotherapy significantly improved prognosis compared with follow-up (5-year OS: 83.3 vs. 41.7%, *P* = 0.047). For patients with distant relapse, consolidative AHSCT after salvage chemotherapy significantly prolonged survival compared with follow-up (2-year OS: 100.0 vs. 20.0%, *P* = 0.004). Patients without consolidative treatment after response to salvage chemotherapy exhibited a comparable survival to those who experienced stable or progressive disease after chemotherapy. Asparaginase (ASP)-containing salvage chemotherapy failed to confer a survival advantage over ASP-absent chemotherapy (5-year OS: 44.2 vs. 39.3%, *P* = 0.369). In conclusion, consolidative RT or AHSCT improved prognosis in patients with relapsed NKTCL who responded to initial salvage chemotherapy, and the role of ASP in salvage chemotherapy requires further exploration in prospective studies.

Extranodal natural killer/T-cell lymphoma (NKTCL) is a distinct lymphoid malignancy in the World Health Organization (WHO) classification^1^,^2^,^3^,^4^. This disease is commonly observed in young males, diagnosed at an early stage, and exhibits a close association with Epstein–Barr virus (EBV) infection^3^,^4^. Due to its rarity, the optimal treatment strategy has yet to be established. Radiotherapy (RT) has been well accepted as the primary treatment for early-stage disease^5^,^6^,^7^ and is also beneficial in select cases of advanced-stage disease^8^. Chemotherapeutic regimens based on anthracycline agents exhibit disappointing efficacy for NKTCL^9^,^10^,^11^,^12^, probably due to the expression of a multidrug-resistant gene in tumor cells^9^. However, novel regimens that contain L-asparaginase (L-ASP) or pegaspargase have elicited promising responses^13^,^14^,^15^,^16^. Autologous and allogeneic hematopoietic stem cell transplantation (HSCT) have been reported as feasible consolidation therapies for high-risk, relapsed, or refractory NKTCL, but whether these approaches are associated with a definitive survival benefit remains controversial^17^,^18^,^19^,^20^,^21^,^22^.

Although approximately 70–90% of early-stage and 15–65% of advanced-stage patients achieve complete remission (CR) after primary therapy^5^,^8^,^10^,^11^,^12^,^13^,^23^,^24^,^25^, a proportion of them eventually experience relapse. The relapse rate of NKTCL varies from 25% to 60%, depending on the treatment modality^26^,^27^,^28^,^29^,^30^,^31^. Due to the rarity of relapsed cases, most studies report patients with relapsed disease together with those who had primary refractory disease or even newly diagnosed advanced-stage disease, which may cause inappropriate interpretation and extrapolation of treatment outcomes^13^,^14^,^15^,^32^,^33^,^34^,^35^. To date, very few results focusing exclusively on the treatment of relapsed NKTCL are available. Two previous studies have reported the benefits of salvage RT for recurrent NKTCL, but the roles of salvage chemotherapy and HSCT remain to be discovered^36^,^37^. In addition, the optimal management of patients with different patterns of relapse, especially those with distant recurrence, remains largely unknown. In this study, we evaluated the outcomes of salvage treatment in a relatively large cohort of patients with relapsed NKTCL, explored the effects of consolidative RT and autologous hematopoietic stem cell transplantation (AHSCT), and compared the efficacy of asparaginase (ASP)-containing and ASP-absent chemotherapy regimens.

## Methods

### Patient selection and evaluation

The inclusion criteria for this study were as follows: (1) pathologically confirmed NKTCL at initial diagnosis according to the WHO classification of lymphomas^3^,^4^; (2) achieved CR after primary treatment according to the International Working Group Recommendations for Response Criteria for non-Hodgkin’s lymphoma^38^,^39^; (3) pathologically or clinically diagnosed relapse; and (4) complete follow-up data. The exclusion criteria included the following: (1) experienced relapse within one month after initial CR; and (2) received best supportive care alone after relapse without anti-tumor therapy. Fifty-six patients with relapsed NKTCL between 2001 and 2013 were included in the analysis, 41 (73.2%) of whom had pathologically confirmed relapse and 15 (26.8%) of whom had clinically diagnosed relapse. Informed consent for the collection of medical information was obtained from all patients at their first visit. All procedures performed in this study were in accordance with the ethical standards of the institutional research committee and with the 1964 Helsinki Declaration and its later amendments or comparable ethical standards. The study protocol was approved by the ethics committee of Sun Yat-sen University Cancer Center.

Clinical evaluations at initial presentation and relapse were performed as previously reported^8^,^16^. Clinical staging of the tumors was performed according to the Ann Arbor system. The International Prognostic Index (IPI) and the natural killer/T-cell lymphoma prognostic index (NKPI) were calculated for all patients at initial presentation^28^,^40^. The primary sites of disease were classified into the upper aerodigestive tract (UAT) and the extra-upper aerodigestive tract (EUAT) as previously reported^41^. Locoregional relapse (LR) was defined as tumor recurrence at the primary site and/or regional lymph nodes, and distant relapse (DR) was defined as tumor relapse at locations other than the primary site or regional lymph nodes.

### Treatment and response evaluation

At initial presentation, the first-line treatment was induction chemotherapy followed by involved-field radiotherapy (IFRT) for patients with early-stage disease (Ann Arbor stage I/II), and chemotherapy alone for those with advanced stage disease (Ann Arbor stage III/IV). Chemotherapy was the initial salvage treatment after relapse. Patients who achieved CR or partial remission (PR) after initial chemotherapy could receive RT or autologous hematopoietic stem cell transplantation (AHSCT) as consolidation therapy, depending on the patient’s physical status and the pattern of recurrence (LR or DR) at the discretion of the treating physician. Chemotherapy regimens varied during the study period according to the treating physicians but were categorized as ASP-containing or ASP-absent regimens, depending on whether L-ASP or pegaspargase was incorporated. Treatment response was evaluated after every two cycles of salvage chemotherapy, before and after consolidative RT or AHSCT (if received), and at the end of salvage treatment, in accordance with the International Working Group Recommendations for Response Criteria for non-Hodgkin’s lymphoma^38^,^39^.

### Statistical analysis

The duration of CR after first-line treatment was defined as the time from the documentation of an initial CR to relapse. Overall survival (OS) was measured from initial diagnosis to death due to any cause or the most recent follow-up. OS after relapse was calculated from the first recurrence to death due to any cause or the most recent follow-up. Survival data were calculated using the Kaplan-Meier method and compared using a log-rank test. Baseline characteristics and treatment response were compared using the Chi-squared test or Fisher’s exact test. A two-sided P value < 0.05 was considered statistically significant. The statistical analysis was performed using SPSS version 17.0 software (SPSS, Chicago, IL, USA).

## Results

### Clinical characteristics and treatment at initial presentation

The clinical characteristics at initial diagnosis and first-line treatment are presented in Table 1. Overall, the cohort exhibited low-risk clinical features. The median age of the cohort at presentation was 38 years (range 9–77 years), and 89.3% of the patients were ≤60 years old. Most of the patients presented with early-stage disease (92.9%), primary disease originating from UAT (82.1%), and a good performance status (Eastern Cooperative Oncology Group [ECOG] score of 0–1 in 91.1% of the patients). The IPI was scored as 0–1 in 87.5% of the patients, and the NKPI score was 0–1 in 69.6% of the patients.

Most of the patients (51, 91.1%) received chemotherapy plus RT as their first-line treatment, whereas four (7.1%) received chemotherapy alone, and one (1.8%) received RT alone. RT was administered at a median dose of 50.4 Gy (range 24.0–68.0 Gy), with 75.0% of the patients receiving a dose of ≥50.0 Gy. ASP-containing and ASP-absent regimens were administered as the first-line chemotherapy to 12 patients (21.8%) and 43 patients (78.2%), respectively. The median number of chemotherapy cycles was 5 (range 1–10), and 72.0% of the patients received ≥4 cycles.

### Clinical characteristics at relapse

As shown in Table 2, the median duration of CR was 8.9 months (range 1.8–84.5 months). Approximately half of the patients (55.4%) experienced relapse within 12 months after the initial CR. Patterns of relapse included LR alone in 23 (41.1%) patients, DR alone in 9 (16.1%) patients, and combined LR and DR in 24 (42.9%) patients. B symptoms and an elevated LDH level were observed in 42.9% and 39.3% of the patients, respectively. Most of the patients exhibited a good performance status (ECOG score 0–1: 82.1%) at relapse.

### Salvage treatment

Salvage treatment after relapse included chemotherapy alone (32 cases, 57.1%), chemotherapy followed by RT (16 cases, 28.6%), and chemotherapy followed by AHSCT (8 cases, 14.3%). As presented in Table 2, among the patients with LR alone, salvage treatment included chemotherapy alone, chemotherapy followed by RT, and chemotherapy followed by AHSCT in 9 (39.1%), 13 (56.1%), and 1 (4.3%) patients, respectively. Of the patients with DR ± LR, chemotherapy alone, chemotherapy followed by RT, and chemotherapy followed by AHSCT were administered to 23 (69.7%), 3 (9.1%), and 7 (21.2%) patients, respectively. Overall, CR, PR, stable disease (SD), and progressive disease (PD) were observed after salvage treatment in 51.8%, 12.5%, 1.8%, and 33.9% of the patients, respectively.

Salvage chemotherapeutic regimens included pegaspargase or L-ASP/gemcitabine/oxaliplatin (P-Gemox/GELOX, 13 cases, 23.2%), ifosfamide/methotrexate/etoposide (IMVP-16, 12 cases, 21.4%), etoposide/doxorubicin/vincristine/cyclophosphamide/prednisone (EPOCH, 7 cases, 12.5%), dexamethasone/ifosfamide/cisplatin/etoposide (DICE, 4 cases, 7.1%), gemcitabine/vinorelbine/doxorubicin (GND, 4 cases, 7.1%), and miscellaneous regimens (16 cases, 28.6%). ASP-containing and ASP-absent regimens were administered to 21 (37.5%) and 35 patients (62.5%), respectively. The median number of chemotherapy cycles was 4 (range 1–9).

Of the 16 patients who received RT after salvage chemotherapy, the pre-RT response was CR/PR in 15 patients and SD in one patient. All of the patients had received RT during first-line treatment. The median radiation dose was 50.0 Gy (range 36.8~60.0 Gy). The radiation field covered all sites of recurrence with adequate margins. RT techniques included conventional two-dimensional RT (9 cases), intensity-modulated RT (IMRT, 4 cases), and three-dimensional conformal RT (3D-CRT, 3 cases).

Of the 8 patients receiving AHSCT after salvage chemotherapy, the pre-transplant response was CR in 7 and PR in one patient. Salvage chemotherapy before AHSCT included IMVP-16 (2 cases), EPOCH (2 cases), SMILE, GND, GELOX, and L-ASP + vincristine + dexamethasone (1 cases for each regimen). Conditioning chemotherapy was BEAM (carmustine, etoposide, cytarabine and melphalan) in 7 patients and GemBuMel (gemcitabine, busulfan and melphalan) in one patient.

### Survival and prognostic factors after relapse

**Figure 1:**
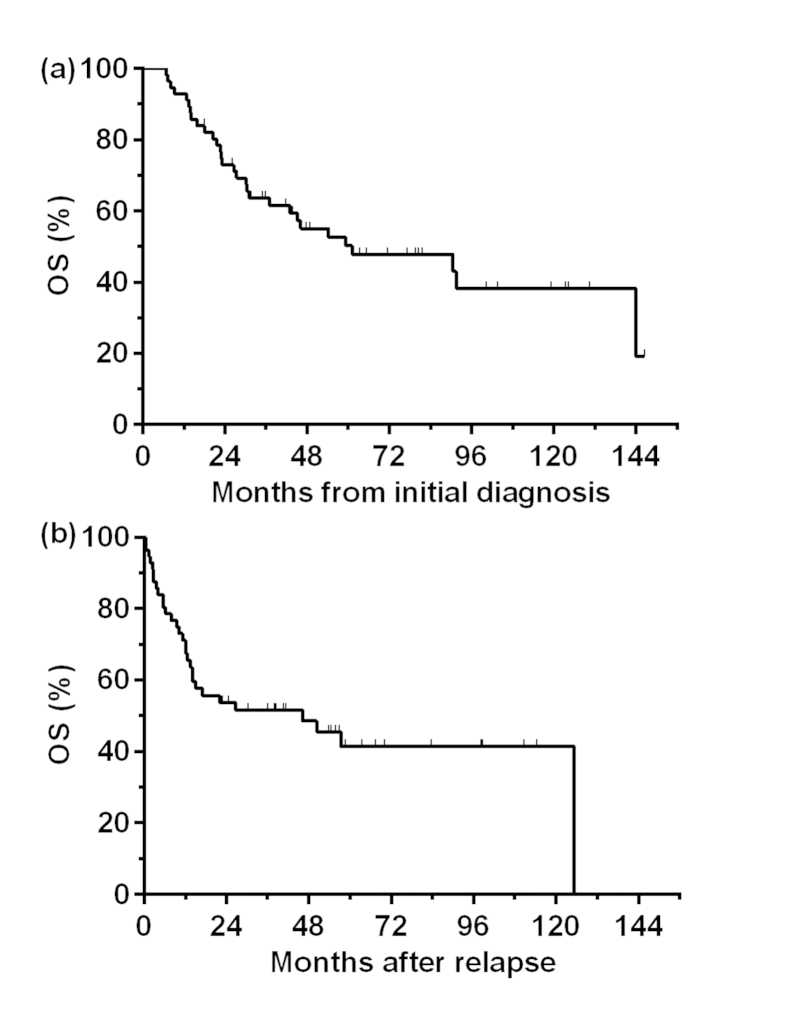
Overall survival (OS) after initial diagnosis **(a)** or after recurrence **(b)** in all patients with relapsed NK/T cell lymphoma.

The median follow-up time for the surviving patients was 74.3 months (range 17.8–146.5 months). At the time of last follow-up, 30 (53.6%) patients had died of lymphoma (*n* = 29) or treatment-related complications (*n* = 1). For the entire cohort, the 5-year OS after initial diagnosis was 50.3%, with a median survival of 61.0 months (Fig. 1a). The 5-year OS after relapse was 41.4%, with a median survival of 46.1 months (Fig. 1b).

**Figure 2:**
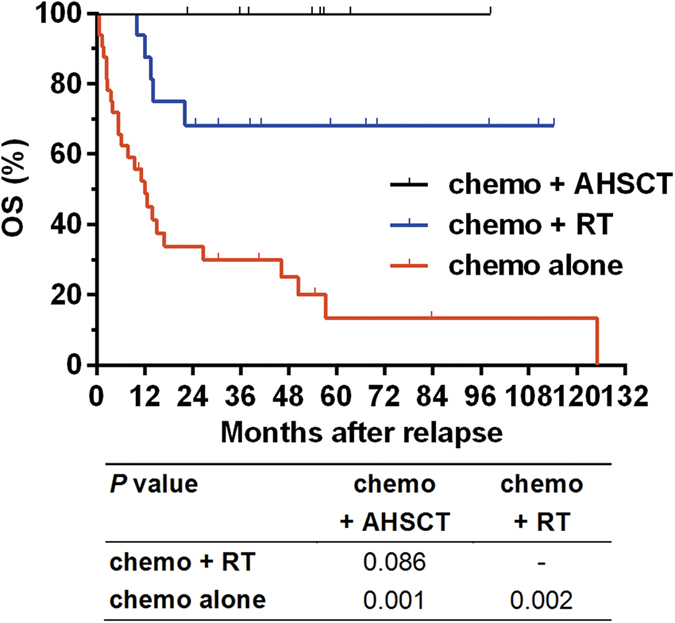
Overall survival (OS) after relapse in patients with relapsed NK/T cell lymphoma who received chemotherapy followed by autologous hematopoietic stem cell transplantation (AHSCT), chemotherapy followed by radiotherapy (RT), or chemotherapy alone.

Survival after relapse significantly differed among patients receiving different salvage treatment modalities. The 5-year OS after relapse was 100.0%, 68.2%, and 13.3% in patients who received chemotherapy followed by AHSCT, chemotherapy followed by RT, and chemotherapy alone, respectively (*P* < 0.001, Fig. 2). Patients who achieved CR after salvage treatment exhibited a substantially prolonged OS after relapse compared with those who failed to achieve CR (5-year OS after relapse: 74.7% vs. 7.8%, *P* < 0.001).

In a univariate analysis, patients with LR alone exhibited a significantly better prognosis than those with DR ± LR (60.9 vs. 27.0%, *P* = 0.016). A duration of CR ≥12 months was associated with a marginally better 5-year OS (50.8 vs. 32.3%, *P* = 0.056). Other predictors of a better 5-year OS after relapse included age ≤60 years (46.2% vs. 0.0% for age >60, *P* = 0.003) and ECOG score of 0–1 (50.2% vs. 10.0% for ECOG ≥2, *P* < 0.001). B symptoms, LDH level, number of extranodal sites, and sex had no significant impact on survival after relapse. Multivariate analysis was not performed due to the small number of events in this cohort 42.

### Salvage treatment and survival of patients with locoregional relapse alone

**Figure 3:**
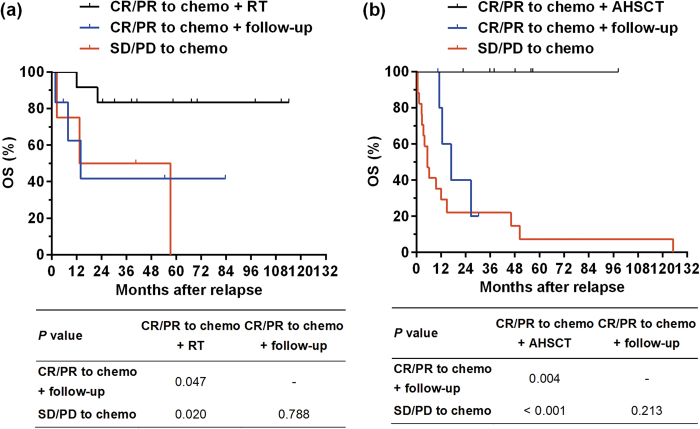
(**a**) Overall survival (OS) after relapse in patients with locoregional relapse alone who received consolidative radiotherapy (RT, the black line) or follow-up (the blue line) after achieving complete or partial remission (CR/PR) after salvage chemotherapy and who exhibited stable or progressive disease (SD/PD) after salvage chemotherapy (red line). (**b**) OS after relapse in patients with distant relapse who received autologous hematopoietic stem cell transplantation (AHSCT, black line) or follow-up (blue line) after exhibiting CR/PR after salvage chemotherapy and those who had SD/PD after salvage chemotherapy (red line).

Of the 23 patients with LR alone, one received AHSCT after exhibiting a CR to salvage chemotherapy (this patient lived without lymphoma for 63 months). The remaining 22 patients were classified into three groups: patients who achieved CR/PR after salvage chemotherapy and then received consolidative RT (group A, 12 patients) or follow-up (group B, 6 patients) and patients who experienced SD/PD after salvage chemotherapy (group C, 4 patients). Clinical characteristics at relapse, first-line treatment, and salvage chemotherapeutic regimens were comparable between patients who received consolidative RT (group A) or follow-up (group B, Table 3). Consolidative RT significantly improved 5-year OS after relapse compared with follow-up (83.3 vs. 41.7%, *P* = 0.047, Fig. 3a). However, survival after relapse was comparable between patients who received follow-up after CR/PR to salvage chemotherapy (group B) and patients with SD/PD to salvage chemotherapy (group C, *P* = 0.788, Fig. 3a).

### Salvage treatment and survival of patients with distant ± locoregional relapse

Of the 33 patients with DR ± LR, three received consolidative RT after achieving CR/PR to salvage chemotherapy, one of whom was alive without lymphoma at 14 months and two of whom died of lymphoma (at 10 and 14 months). The remaining 30 patients were classified into three groups: patients who achieved CR/PR after salvage chemotherapy and then received AHSCT as consolidative therapy (group A, seven patients) or who received follow-up (group B, six patients) and patients who experienced SD/PD after salvage chemotherapy (group C, 17 patients). As shown in Table 3, the clinical characteristics at relapse, first-line treatment, and salvage chemotherapeutic regimens were comparable between patients who received consolidative AHSCT (group A) or follow-up (group B). Marginally more patients in group A achieved CR after chemotherapy; however, this difference did not reach statistical significance (100 vs. 50%, *P* = 0.070). Compared with follow-up, AHSCT after response to salvage chemotherapy significantly improved 2-year OS after relapse (100.0 vs. 20.0%, *P* = 0.004, Fig. 3b). All patients in the AHSCT group survived without further recurrence at a median follow-up of 48.1 months. However, survival after relapse was comparable between patients who received follow-up after CR/PR to salvage chemotherapy (group B) and patients who experienced SD/PD after salvage chemotherapy (group C, *P* = 0.213, Fig. 3b).

### Role of asparaginase in salvage treatment

**Figure 4:**
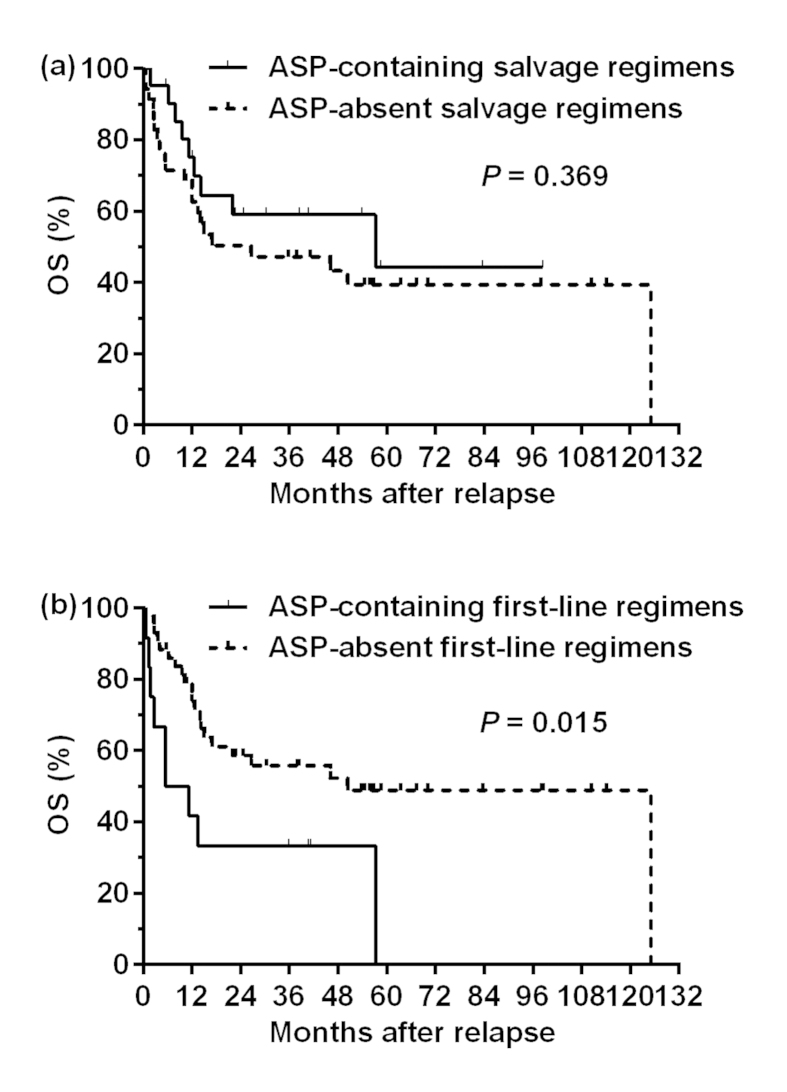
Overall survival (OS) after relapse in patients who received asparaginase (ASP)-containing or ASP-absent regimens (**a**) as salvage chemotherapy or (**b**) as first-line chemotherapy.

After initial salvage chemotherapy, CR, PR, SD, and PD were observed in 37.5%, 25.0%, 3.6%, and 33.9% of the patients, respectively. The CR rate for ASP-containing regimens was significantly higher than that for ASP-absent regimens (66.7 vs. 20.0%, *P* < 0.001), but the overall response rates (ORR: CR + PR) were not significantly different (76.2 vs. 54.3%, *P* = 0.101). Patients who received ASP-containing or ASP-absent regimens as salvage chemotherapy exhibited comparable survival (5-year OS after relapse: 44.2 vs. 39.3%, *P* = 0.369, Fig. 4a). 19.0% of patients in the ASP-containing group and 23.5% of the patients in the ASP-absent group at relapse received ASP during their first-line chemotherapy (*P* = 0.750). Responses to chemotherapy during the first-line treatment were comparable between the ASP-containing and ASP-absent groups at relapse (CR + PR: 81.3 vs. 75.0%, *P* = 0.724). However, there were significantly more patients with an ECOG score ≥2 in the ASP-absent group than in the ASP-containing group (25.7 vs. 4.8%, *P* = 0.047, Table 4). Among those who received ASP-containing chemotherapy, response (CR + PR: 72.7 vs. 80.0%, *P* = 1.000) and survival (2-year OS after relapse: 70.0 vs. 46.7%, *P* = 0.488) were not significantly different between the patients who received L-ASP and those who received pegaspargase.

We also assessed the impact of first-line chemotherapeutic regimens on survival after relapse. During the first-line treatment, 43 (78.6%) and 12 (21.4%) patients received ASP-absent and ASP-containing regimens, respectively, as their induction chemotherapy. As shown in Table 4, no significant difference was observed in the clinical characteristics at relapse, and the first-line and salvage treatment modalities between the two groups. However, patients who received ASP-containing first-line chemotherapy exhibited a substantially worse response to salvage chemotherapy after recurrence compared with those who received ASP-absent first-line chemotherapy (CR + PR: 33.3 vs. 69.8%, *P* = 0.041). Accordingly, patients who received ASP-containing first-line chemotherapy exhibited significantly worse survival than those who received ASP-absent first-line chemotherapy (2-year OS after relapse: 33.3 vs. 58.6%, *P* = 0.015, Fig. 4b).

## Discussion

In this study, we focused exclusively on the treatment modalities and prognosis of patients with relapsed NKTCL. To our knowledge, this is the largest cohort of this subgroup of patients studied to date. Our findings suggest that the overall prognosis of relapsed NKTCL remains dismal, but patients who achieved CR after salvage treatment exhibited significantly better survival after relapse. For patients with locoregional relapse alone who achieved CR/PR to salvage chemotherapy, consolidative RT substantially improved prognosis compared with follow-up. For patients with distant relapse, adding AHSCT after achieving CR/PR to salvage chemotherapy elicited a significantly better survival compared with follow-up alone. Salvage chemotherapeutic regimens incorporating ASP did not exhibit a significant survival advantage than those without ASP. However, patients who received ASP in their first-line chemotherapy exhibited a poorer response to salvage chemotherapy as well as a worse prognosis after relapse compared with those who received ASP-absent first-line chemotherapy.

The first-line treatment of NKTCL has significantly evolved during the last decade. However, the optimal treatment modality after relapse remains poorly defined to date. Due to the rarity of such cases, only retrospective data from two small cohorts are available^36^,^37^. RT has been well established as the primary treatment for early stage disease due to the high radiosensitivity of NKTCL^5^,^6^,^7^. Patients with relapsed disease may also benefit from RT. In two previous studies and in the present study, RT at a median dose of 40–50 Gy with or without chemotherapy yielded a 5-year OS of 62.5–83.3% in patients with localized or locoregional relapse^36^,^37^. More importantly, our and Zhao’s studies consistently demonstrated that RT with or without chemotherapy produced markedly better survival than chemotherapy alone in patients with locoregional relapse^36^. In addition, most of those patients receiving re-irradiation in our and Zhao’s cohorts had previously received RT in the first-line treatment, indicating that recurrent NKTCL remains radiosensitive. Considering the radiosensitivity and favorable prognosis, it is reasonable to consider RT at a median dose of 40–50 Gy as an integral part of salvage treatment in patients with locoregionally recurrent NKTCL.

Several studies have investigated the role of HSCT in newly diagnosed or relapsed/refractory NKTCL patients and have reported that AHSCT may be a feasible therapeutic option in selected patients (e.g., in CR before transplant or with advanced stage, poor prognostic index, or high EBV-DNA copy number)^17^,^18^,^19^,^20^,^43^. However, those studies have limitations due to their small sample sizes, inconsistent indications for AHSCT, and heterogeneous conditioning regimens. In addition, little evidence is available to demonstrate a definitive survival advantage of HSCT over follow-up after remission. In a relatively large-scale matched controlled analysis, Lee *et al*. revealed that AHSCT significantly improved disease-specific survival in patients who achieved CR after primary or salvage treatment or in those with a high NKPI score compared with the historical control group who did not received AHSCT^17^. Similar results were also observed in other smaller cohorts, as reported by Kim *et al*. and Au *et al*.^19^,^44^. However, the historically matched comparison in those studies may inevitably lead to selection bias. In the present study, the indication and conditioning regimen (CR after salvage chemotherapy and BEAM in 7 of 8 patients) for AHSCT were relatively uniform. In patients with distant relapse who achieved CR/PR after initial salvage chemotherapy, AHSCT as consolidative therapy substantially improved prognosis compared with follow-up. All of the seven patients who received AHSCT survived at a median follow-up of 48.1 months without a second relapse or treatment-related mortality, which was a very encouraging result considering the extensive relapse in those patients. A possible reason for this result is that most of these patients had attained CR before transplantation, which has been reported to be a powerful prognostic factor for better survival after HSCT in previous studies^17^,^19^,^45^,^46^,^47^. Additionally, due to the long study period covered in our report, the chemotherapeutic regimens showed a great variety among the patients, including those who received AHSCT. However, the heterogeneous chemotherapy received before AHSCT suggested that the excellent outcome observed in the AHSCT group might be attributed to the transplantation itself, rather than some specific induction chemotherapeutic regimens.

Overexpression of a multidrug-resistant gene was most likely the reason that NKTCL exhibits disappointing responses to anthracycline-based chemotherapeutic regimens (such as CHOP and EPOCH) as first-line treatments^9^,^10^,^11^,^12^. Asparaginase (L-asparaginase or pegaspargase) exhibits a unique antitumor mechanism via hydrolyzing serum asparagine. NKTCL cells heavily depend on exogenous asparagine and are therefore highly susceptible to asparaginase^48^. For relapsed or refractory patients, chemotherapeutic regimens without ASP yielded a CR rate of only 13–37% and an ORR rate of 36–52%^34^,^36^,^49^. In Zhang’s study, CHOP or EPOCH regimens failed to improve prognosis in patients with localized relapse and resulted in a dismal survival in disseminated recurrent cases (1-year OS was 0%)^37^. In contrast, ASP-containing chemotherapy yielded a CR rate of 45–61% and an ORR rate of 70–88% in patients with relapsed or refractory NKTCL^13^,^14^,^15^,^33^,^34^,^35^,^50^,^51^. In a study of 42 patients with stage IV, relapsed, or refractory NKTCL, the SMILE (L-asparaginase, methotrexate, ifosfamide, etoposide, and dexamethasone) regimen significantly improved response rates and survival compared with CHOP^34^. However, salvage chemotherapy with ASP-containing or ASP-absent regimens produced comparable ORR rates and survival in our cohort of relapsed patients, although the ASP-containing regimens were associated with a higher CR rate. Several explanations may account for this discrepancy. First, heterogeneity existed with respect to the chemotherapy regimens used in our cohort. Second, a relatively high proportion (42.9%) of patients in our cohort received consolidative RT or AHSCT after salvage chemotherapy, which may have attenuated the difference in efficacy between those chemotherapeutic regimens. In addition, clinical characteristics also differed between the ASP-containing and ASP-absent groups, with significantly more patients with poor physical status in the ASP-absent group. It is noteworthy that the continuous improvement of first-line chemotherapy over the study period might result in more chemoresistant relapse and confounded the comparison between the ASP-containing and ASP-absent chemotherapy at relapse. However, the first-line chemotherapeutic regimens and response were comparable between the ASP-containing and ASP-absent groups after relapse according to our data. Therefore, we consider the efficacy of first-line chemotherapy might not be a major confounding factor to the comparison between ASP-containing and ASP-absent regimens at relapse. This question remains open, and a prospective study consisting of more patients is required to assess the role of ASP in salvage treatment. Another noteworthy finding in our study was that patients who received ASP-containing chemotherapy as their first-line treatment exhibited a poorer response to salvage chemotherapy and worse prognosis after relapse compared with those who received ASP-absent first-line chemotherapy. This finding indicates that effective first-line therapy may result in more chemoresistant relapsed diseases, and more effective drugs and regimens are required for patients who experience relapse after ASP-containing first-line chemotherapy.

In the present study, chemotherapy as initial salvage treatment for relapsed NKTCL achieved an ORR of 62.5%, but patients receiving chemotherapy alone exhibited a 5-year OS of only 13.3%. Without subsequent consolidative therapy, patients, with either locoregional or distant relapse, who achieved CR/PR after salvage chemotherapy exhibited a survival comparable to those who experienced SD/PD. Therefore, chemotherapy alone is inadequate for relapsed NKTCL, and appropriate consolidative therapies according to the pattern of relapse are indispensable.

Consistent with the prior studies of Zhang *et al*. and Zhao *et al*., the overall prognosis of our relapsed cohort remained relatively poor, with a 5-year OS of 41.4% after relapse (40.0% by Zhao *et al*. and 37.8% by Zhang *et al*.)^36^,^37^. Notably, most of the patients in our study had been heavily treated in the first-line setting, with 91% receiving combined chemotherapy and RT, 72% receiving more than four cycles of chemotherapy, and 75% receiving RT of ≥50 Gy, making salvage treatment after relapse challenging. However, patients who attained CR after salvage treatment (51.8%) were nonetheless able to achieve an encouraging survival rate after relapse (5-year OS of 74.7% in our cohort, and 2-year OS of 86.0% in Zhao’s cohort)^36^. The plateau observed in survival curves indicated that a proportion of relapsed cases remained potentially curable with appropriate salvage treatment. Other predictors of OS observed in our cohort, including pattern of relapse (LR or DR) and duration of CR, were also been observed by Zhao *et al*.^36^.

The limitations of this study are its retrospective nature and the small sample size. Neither the role of consolidative RT in patients with distant relapse nor the role of AHSCT in patients with locoregional relapse could be determined due to the very limited number of cases. Additionally, we were unable to define whether patients who achieved PR following salvage chemotherapy could benefit from AHSCT due to the limited number of patients. Moreover, we could not perform a multivariate analysis to identify independent prognostic factors in this cohort due to the insufficient number of events.

## Conclusion

The results of the present retrospective analysis indicate that patients with relapsed NKTCL who respond to initial salvage chemotherapy may benefit from consolidative RT or AHSCT, depending on their pattern of relapse. Incorporating ASP into salvage chemotherapy failed to improve survival after relapse. However, patients who received ASP during their first-line chemotherapy exhibited a poorer response to salvage chemotherapy and worse prognosis after relapse. Patients with relapsed NKTCL were potentially curable with appropriate salvage chemotherapy followed by consolidative treatment, which should be further explored in studies using prospective designs and larger sample sizes.

## Additional Information

**How to cite this article**: Nie, M. *et al*. Consolidative treatment after salvage chemotherapy improves prognosis in patients with relapsed extranodal natural killer/T-cell lymphoma. *Sci. Rep*. **6**, 23996; doi: 10.1038/srep23996 (2016).

## Figures and Tables

**Table 1:** The clinical characteristics and treatment modalities at the initial presentation of the patients with recurrent NK/T-cell lymphoma.

Parameters	*n*(%)	Parameters	*n*(%)
Overall	56 (100)	B symptoms	26 (46.4)
Sex		Elevated LDH	12 (21.4)
Male	36 (64.3)	NKPI	
Female	20 (35.7)	0–1	39 (69.6)
Age (years)		2–4	17 (30.4)
Median (range)	38 (9–77)	First-line treatment	
≤60	50 (89.3)	Chemotherapy alone	4 (7.1)
Primary site		RT alone	1 (1.8)
UAT	46 (82.1)	Chemotherapy and RT	51 (91.1)
EUAT	10 (17.9)	Radiation dose (Gy)	
Ann Arbor stage		Median (range)	50.4 (24.0–68.0)
I–II	52 (92.9)	<50 Gy	13 (25.0)
III–IV	4 (7.1)	≥50 Gy	39 (75.0)
ECOG score		Chemotherapy regimens	
0–1	51 (91.1)	ASP-absent	43 (78.2)
≥2	5 (8.9)	ASP-containing	12 (21.8)
IPI		Chemotherapy cycles	
0–1	49 (87.5)	Median (range)	5 (1–10)
2–3	7 (12.5)	<4	14 (28.0)
4–5	0 (0.0)	≥4	36 (72.0)

Abbreviations: ASP: asparaginase; ECOG: Eastern Cooperative Oncology Group; EUAT: extra-upper aerodigestive tract; IPI: International Prognostic Index; LDH: lactate dehydrogenase; NKPI: natural killer/T-cell lymphoma prognostic index; RT: radiotherapy; UAT: upper aerodigestive tract.

**Table 2:** The clinical characteristics at relapse of patients with recurrent NK/T-cell lymphoma.

Parameters	*n*(%)	Parameters	*n*(%)
Overall	56 (100)	B symptoms	24 (42.9)
Age (years)		Elevated LDH	22 (39.3)
Median (range)	39 (10–79)	Duration of CR after first-line therapy (months)	
≤60	49 (87.5)	Median (range)	8.9 (1.8–84.5)
Pattern of relapse		<12 months	31 (55.4)
Locoregional alone	23 (41.1)	≥12 months	25 (44.6)
Distant alone	9 (16.1)	Salvage treatment of locoregional relapse alone (*n* = 23)	
Locoregional + distant	24 (42.9)	CT alone	9 (39.1)
Extranodal involvement		CT followed by RT	13 (56.5)
0–1 site	35 (62.5)	CT followed by AHSCT	1 (4.3)
≥2 sites	21 (37.5)	Salvage treatment of distant ± locoregional relapse (*n* = 33)	
ECOG score		CT alone	23 (69.7)
0–1	46 (82.1)	CT followed by RT	3 (9.1)
≥2	10 (17.9)	CT followed by AHSCT	7 (21.2)

Abbreviations: AHSCT: autologous hematopoietic stem cell transplantation; CR: complete remission; CT: chemotherapy; ECOG: Eastern Cooperative Oncology Group; LDH: lactate dehydrogenase; RT: radiotherapy.

**Table 3:** Clinical characteristics at relapse and salvage treatment in patients who responded to salvage chemotherapy.

Parameters	Patient with locoregional relapse alone			Patients with distant ± locoregional relapse		
	Consolidative RT *n*(%)	Follow-up *n*(%)	*P*value	AHSCT *n*(%)	Follow-up *n*(%)	*P*value
Overall	12 (100)	6 (100)	–	7 (100)	6 (100)	–
Age >60 years	1 (8.3)	3 (50.0)	0.083	0 (0.0)	1 (16.7)	0.462
Pattern of relapse						
Locoregional alone	12 (100)	6 (100)	–	0 (0.0)	0 (0.0)	–
Distant ± locoregional	0 (0.0)	0 (0.0)		7 (100)	6 (100)	
Extranodal involvement ≥2 sites	1 (8.3)	0 (0.0)	1.000	5 (71.4)	2 (33.3)	0.286
B symptoms	4 (33.3)	2 (33.3)	1.000	3 (42.9)	1 (16.7)	0.559
Elevated LDH	3 (25.0)	0 (0.0)	0.515	5 (71.4)	2 (33.3)	0.286
ECOG score ≥2	0 (0.0)	0 (0.0)	–	0 (0.0)	0 (0.0)	–
First-line treatment						
CT alone	0 (0.0)	2 (33.3)	0.098	0 (0.0)	0 (0.0)	–
RT ± CT	12 (100.0)	4 (66.7)		7 (100)	6 (100)	
Salvage CT						
ASP-containing	5 (41.7)	4 (66.7)	0.620	3 (42.9)	3 (50.0)	1.000
ASP-absent	7 (58.3)	2 (33.3)		4 (57.1)	3 (50.0)	
Response to salvage CT						
CR	5 (41.7)	3 (50.0)	1.000	7 (100)	3 (50.0)	0.070
PR	7 (58.3)	3 (50.0)		0 (0.0)	3 (50.0)	

Abbreviations: AHSCT: autologous hematopoietic stem cell transplantation; ASP: asparaginase; CR: complete remission; CT: chemotherapy; ECOG: Eastern Cooperative Oncology Group; IPI: International Prognostic Index; LDH: lactate dehydrogenase; NKPI: natural killer/T-cell lymphoma prognostic index; PR: partial remission; RT: radiotherapy.

**Table 4:** Clinical characteristics and treatment at relapse in patients who received ASP-absent or ASP-containing regimens as the first-line or salvage chemotherapy.

Parameters	First-line chemotherapy			Salvage chemotherapy		
	ASP-absent *n*(%)	ASP-containing *n*(%)	*P*value	ASP-absent *n*(%)	ASP-containing *n*(%)	*P*value
Overall	43 (100)	12 (100)	–	35 (100)	21 (100)	–
Age >60 years	6 (14.0)	1 (8.3)	1.000	4 (11.4)	3 (14.3)	0.754
Pattern of relapse						
Locoregional alone	18 (41.9)	5 (41.7)	0.990	12 (34.4)	11 (52.4)	0.183
Distant ± locoregional	25 (58.1)	7 (58.3)		23 (65.7)	10 (47.6)	
Extranodal involvement ≥2 sites	16 (37.2)	5 (41.7)	0.779	11 (31.4)	10 (47.6)	0.264
B symptoms	16 (37.2)	8 (66.7)	0.101	17 (48.6)	7 (33.3)	0.265
Elevated LDH	16 (37.2)	6 (50.0)	0.424	14 (40.0)	8 (38.1)	0.888
ECOG score ≥2	6 (14.0)	4 (33.3)	0.199	9 (25.7)	1 (4.8)	0.047
First-line treatment						
CT alone	2 (4.7)	2 (16.7)	0.204	2 (5.7)	2 (9.5)	0.626
RT ± CT	41 (95.3)	10 (83.3)		33 (94.3)	19 (90.5)	
Salvage treatment						
CT alone	23 (53.5)	9 (75.0)	0.409	20 (57.4)	12 (57.1)	1.000
CT + AHSCT	7 (16.3)	1 (8.3)		5 (14.3)	3 (14.3)	
CT + RT	13 (30.2)	2 (16.7)		10 (28.6)	6 (28.6)	
Salvage CT						
ASP-containing	17 (39.5)	4 (33.3)	0.750	–	–	–
ASP-absent	26 (60.5)	8 (66.7)		–	–	

Abbreviations: AHSCT: autologous hematopoietic stem cell transplantation; ASP: asparaginase; CR: complete remission; CT: chemotherapy; ECOG: Eastern Cooperative Oncology Group; IPI: International Prognostic Index; LDH: lactate dehydrogenase; NKPI: natural killer/T-cell lymphoma prognostic index; RT: radiotherapy.
